# Assessing the Impact of a Global Health Fellowship on Pharmacists’ Leadership Skills and Consideration of Benefits to the National Health Service (NHS) in the United Kingdom

**DOI:** 10.3390/healthcare9070890

**Published:** 2021-07-15

**Authors:** Claire Brandish, Frances Garraghan, Bee Yean Ng, Kate Russell-Hobbs, Omotayo Olaoye, Diane Ashiru-Oredope

**Affiliations:** 1 Pharmacy, Buckinghamshire Healthcare NHS Trust, Aylesbury HP21 8AL, UK; claire.brandish@nhs.net (C.B.); beeyean.ng@nhs.net (B.Y.N.); kate.russellhobbs@nhs.net (K.R.-H.); 2 Pharmacy, Manchester University NHS Foundation Trust, Manchester M13 9PL, UK; frances.garraghan@mft.nhs.uk; 3 The Commonwealth Pharmacists Association (CPA), London E1W 1AW, UK; omotayo.olaoye@commonwealthpharmacy.org

**Keywords:** Commonwealth Partnerships for Antimicrobial Stewardship (CwPAMS), National Health Service (NHS), Chief Pharmaceutical Officer’s Global Health Fellowship, CPhOGH Fellows, CwPAMS, pharmacy, fellowship, health partnerships, antimicrobial resistance (AMR), global health, leadership

## Abstract

Antimicrobial resistance (AMR) poses a global, public health concern that affects humans, animals and the environment. The UK Fleming Fund’s Commonwealth Partnerships for Antimicrobial Stewardship (CwPAMS) scheme aimed to support antimicrobial stewardship initiatives to tackle AMR through a health partnership model that utilises volunteers. There is evidence to indicate that NHS staff participating in international health projects develop leadership skills. Running in parallel with the CwPAMS scheme was the first Chief Pharmaceutical Officer’s Global Health (CPhOGH) Fellowship for pharmacists in the UK. In this manuscript, we evaluate the impact, if any, of participation in the CwPAMS scheme and the CPhOGH Fellowship, particularly in relation to leadership skills, and consider if there are demonstrable benefits for the NHS. The 16 CPhOGH Fellows were invited to complete anonymised baseline and post-Fellowship self-assessment. This considered the impact of the Fellowship on personal, professional and leadership development. Senior colleagues were invited to provide insights into how the Fellows had performed over the course of the Fellowship. All Fellows responded to both the pre- and post-Fellowship questionnaires with a return of 100% (16/16) response rate. There was a significant improvement in Fellows’ perception of their confidence, teaching abilities, understanding of behaviour change, management and communication skills. However, there was no change in the Fellows’ attitude to work. Feedback was received from 26 senior colleagues for 14 of the CPhOGH Fellows. Overall, senior colleagues considered CPhOGH Fellows to progress from proficient/established competencies to strong/excellent when using the national pharmacy Peer Assessment Tool and NHS Healthcare Leadership Model. The majority (88%) of senior colleagues would recommend the Fellowship to other pharmacists. The analysis of the data provided suggests that this CPhOGH Fellowship led to the upskilling of more confident, motivated pharmacist leaders with a passion for global health. This supports the NHS’s long-term plan “to strengthen and support good compassionate and diverse leadership at all levels”. Constructive feedback was received for improvements to the Fellowship. Job satisfaction and motivation improved, with seven CPhOGH Fellows reporting a change in job role and five receiving a promotion.

## 1. Introduction

### 1.1. A Health Partnerships Approach to Supporting Antimicrobial Stewardship in Four African Countries: The Commonwealth Partnerships for Antimicrobial Stewardship (CwPAMS)

Antimicrobial resistance (AMR) poses a global, public health concern that affects humans, animals and the environment [1]. In 2015, the World Health Assembly endorsed a global action plan to tackle the worldwide problem of AMR [1]. This plan encourages the use of a One Health, multi-sectoral approach, and calls for collaboration and coordination locally, and globally. The UK 5-year AMR Action Plan [2], the 20-year Vision for AMR [3] and the NHS Long-Term Plan [4], align with these intentions and build on existing achievements. Engagement and leadership are required at all levels to support progress internationally and to achieve the ambitions for containment and control of AMR globally. Sustained and focused efforts are required to minimise infections, provide safe and effective care to patients and raise awareness of AMR. Pharmacists have been at the forefront of successful antimicrobial stewardship (AMS) programmes across the NHS and, as such, are well placed to support further developments [5,6].

In recognition of the need to create leaders within this field, the Commonwealth Pharmacists Association (CPA) and the Tropical Health and Education Trust (THET) received UK Aid funds through the Department of Health and Social Care’s Fleming Fund, for the pioneering Commonwealth Partnerships for Antimicrobial Stewardship scheme (CwPAMS) in 2019 [7]. A total of 12 health partnerships were formed between multidisciplinary teams from institutions in the UK, including NHS Trusts, together with institutions in Ghana, Tanzania, Uganda, and Zambia. These partnerships were created to allow ideas and knowledge exchange to further develop innovative ways to tackle the problem of AMR and raise awareness, which will mutually benefit the UK and low-to-middle-income countries (LMICs) [8,9]. Success of the CwPAMS projects was dependent on strong leadership and project management within each partnership. Similar qualities are required to facilitate effective approaches for systemwide working in the UK as transitions are made towards Integrated Care Partnership models of care [10].

### 1.2. Pharmacy Leaders and the NHS

Strong and influential leaders across a wide range of healthcare disciplines are essential in undertaking the WHO global AMR action plan [1]. There is evidence to indicate that NHS staff participating in international health projects develop leadership skills essential for influencing change and develop ways of working in the UK [9]. All NHS staff need inclusive leadership skills, which reinforce values and standards of care to drive improvement, leading to the highest quality of patient care [11,12,13,14,15,16]. Accessible leadership development programmes are fundamental to ensure that the NHS workforce is competent in the core leadership domains outlined in the NHS Healthcare Leadership Model [17]. Leadership should be integrated into the training and development offered to NHS staff to ensure good engagement and representation systemwide alongside clinical competencies [11,12].

The national professional body for pharmacists—the Royal Pharmaceutical Society (RPS)—has identified leadership as a key skill required of a pharmacist and has developed frameworks to support this [18]. In addition, the RPS has developed a policy on ‘The pharmacy contribution to antimicrobial stewardship’ and has identified the role that pharmacy leadership has in effective antimicrobial stewardship [19]. Internationally, leadership development and antimicrobial stewardship feature as 2 of the 21 development goals of the International Pharmaceutical Federation (FIP) [20]. Despite the existence of these frameworks and recognition of leadership as a desirable attribute, there are few opportunities available for pharmacists specifically to develop and demonstrate these skills. 

### 1.3. Chief Pharmaceutical Officer’s Global Health (CPhOGH) Fellowship Programme

Health Education England (which exists to provide national leadership and coordination for education and training within the health and public health workforce in England) offer an Improving Global Health (IGH) Fellowship to support the delivery of sustainable improvements in LMICs, whilst developing the transferrable leadership skills of the IGH Fellows to apply on return to the UK [21]. Historically, global health fellowship participants have mainly been doctors and nurses, despite being open to all NHS cadres. The CwPAMS scheme was the first of its kind to mandate that NHS pharmacists be included as essential members of each global health partnership.

Running in parallel with the CwPAMS scheme was the first Chief Pharmaceutical Officer’s Global Health (CPhOGH) Fellowship programme. The CPhOGH scheme was a unique leadership development programme with the aim of cultivating pharmacists as clinical leaders of the future [22]. The development of the fellowship followed a request from Dr Keith Ridge, England’s Chief Pharmaceutical Officer, recognising the positive impact the scheme could have for NHS staff [7]. In addition, the fellowship supported the participants by broadening their knowledge and understanding of global health. The Fellowship was led by CPA and funded by Health Education England (HEE).

The yearlong CPhOGH Fellowship required attendance at an inception workshop, to develop an awareness of skills and behaviours related to the Myers–Briggs Type Indicator (MBTI) questionnaire [23] and NHS Healthcare Leadership Model [17]. The Fellowship also involved completion of the Edward Jenner Professional Leadership Programme [24], a project management module, attendance and engagement in global pharmacy webinars, online action learning sets and responsibility for at least one deliverable within their respective CwPAMS partnership project. Each Fellow was assigned a leadership mentor through HEE’s IGH International Health Fellowship programme [25] for the duration of the Fellowship to discuss the MBTI, reflect on the NHS Healthcare Leadership Model [17] and to provide support and challenge.

In this manuscript, we evaluate the impact, if any, of participation in the CwPAMS scheme and the CPhOGH Fellowship, particularly in relation to leadership skills, and consider if there are demonstrable benefits for the NHS. This will allow understanding of the value of the fellowship and potential future as a personal and professional development opportunity for NHS pharmacists.

## 2. Materials and Methods

Following a selection process, sixteen pharmacists that were involved in CwPAMS partnerships were appointed to join the CPhOGH Fellowship year. These pharmacists were all included in the evaluation of the Fellowship and are referred to as CPhOGH Fellows.

### 2.1. Pre- and Post-Fellowship Self-Assessment by Fellows

The 16 CPhOGH Fellows were invited to complete an online baseline questionnaire (see Supplementary Table S1) designed to capture demographic data and motivations for applying to the CPhOGH Fellowship. Leadership and global health experience was ascertained using a combination of open and closed questions. The questionnaire incorporated the Measuring the Outcomes of Volunteering for Education-Tool (MOVE-iT), a validated tool used to understand the impact of international placements [25]. This questionnaire was reviewed by members of Health Education England and the CPA. For the post-Fellowship evaluation, the baseline questionnaire was reviewed to consider which questions were most relevant and should be repeated in the post-Fellowship questionnaire (see Supplementary Table S2). Additional questions were included to elicit further information on the participants’ leadership skills, development, project management skills and to understand if there were any benefits for the NHS. The pre-CPhOGH Fellows questionnaire consisted of 39 questions and the post-CPhOGH Fellows questionnaire consisted of 45 questions. These comprised free-text responses and statements to be answered according to a 7-pointed Likert scale ranging from “strongly agree” to “strongly disagree” [26]. The survey allowed for qualitative and quantitative analysis of the results.

### 2.2. Assessment by Senior Colleagues

Since this was a programme of leadership and development, the CPhOGH Fellows were invited to seek feedback from at least two senior colleagues who had worked with them prior to starting the Fellowship and for at least a year thereafter. This was in the form of an anonymous online questionnaire to comment on the CPhOGH Fellows’ development and leadership skills (see Supplementary Table S3). A 16-item questionnaire was constructed to gain an understanding of how the CPhOGH Fellows had developed or changed, and how performance was perceived according to their senior peers over the course of the CPhOGH Fellowship. It was based on the NHS Healthcare Leadership Model [17] and the RPS Peer Assessment Tool, adapted and used with permission from the RPS [27]. These were chosen as they are validated, evidence-based models currently used to assess leadership skills in healthcare settings [17,27]. The questionnaire assessed the CPhOGH Fellows on the following dimensions:

- Teamwork;
- Influencing for results;
- Vision, Motivation and capability;
- Inspiring shared purpose;
- Managing change;
- Innovative working and practice.

Within each dimension, senior colleagues were invited to answer a series of questions using a scoring system to indicate their perception of the CPhOGH Fellow’s level of competence before and after the CPhOGH Fellowship [17,27]. The scoring system adopted, ranged from 0 (Unable to comment), 1 (Essential); 2 (Proficient); 3 (Strong) to 4 (Exceptional), which aligns with the RPS Tool [27]. There was a free-text section for each dimension to capture additional feedback and examples (see Supplementary Table S3).

### 2.3. Distribution of Questionnaires

All three questionnaires were hosted on Survey Monkey©. The pre-Fellowship survey was open in June 2019 and CPhOGH Fellows were invited to complete this prior to attending the inception workshop 4–6 July 2019. The post-Fellowship survey and assessment by senior colleagues were open between 9 and 14 August 2020.

### 2.4. Data about Contributions, Achievements, and Communications of CPhOGH Fellows

Using the Fellow’s network, the sixteen CPhOGH Fellows were invited to provide examples of achievements, contributions, and work undertaken to demonstrate the depth and breadth of the experiences gained since being inducted onto the Fellowship. Twitter© and self-reported activities were utilised to capture events and communications using the hashtags #CwPAMS and #CPhOGHFellows to monitor activity. A list of activities was collated and grouped into themes (see Supplementary Tables S4 and S5).

### 2.5. Data Analysis

Some of the data captured in the CPhOGH Fellows’ self-assessment questionnaires were not analysed as they were not considered relevant to this leadership peer review; see Supplementary Tables S1 and S2 for the list of included and excluded questions as well as the rationale for exclusion. All data from the assessment of CPhOGH Fellows’ leadership skills by senior colleagues were included in analysis.

Data were exported to Microsoft Excel© and anonymised before analysis and interpretation. Likert scale responses for components of the MOVE-iT tool, and reflective statements for professional activities and skills undertaken and developed over the CPhOGH Fellowship year were assigned values (7 = Strongly agree to 1 = Strongly disagree) resulting in an aggregate score for each assessed section. The higher the score, the more positive the Fellow’s perception of the assessed components of the MOVE-iT tool and professional development during the Fellowship, respectively.

The Wilcoxon signed rank test was used to evaluate if there was any evidence of differences in average aggregated scores for components of the MOVE-iT tool before and after the Fellowship. Spearman’s correlation test was also used to investigate the evidence of a relationship between Fellows’ previous global health experience and reflective statements for professional activities and skills undertaken and developed over the CPhOGH Fellowship year. Both analyses were conducted using R software. Statistical significance was set at *p* < 0.05. Non-parametric tests (Wilcoxon signed rank test and Spearman’s correlation) were conducted because of the population size and relatively skewed distribution of Fellows’ responses.

Two CPhOGH Fellows reviewed the anonymised, qualitative data independently using thematic analysis to determine the key themes. Non-specific or duplicated quotes were removed, for example “leadership skills “. Where names were used or “he” or “she”, these were changed to “the Fellow” to maintain anonymity.

Ethical approval was not required as per NHS Health Research Authority guidance and the NHS health research decision tool because this was a service evaluation of CPA’s programme of activities to lead the CPhOGH Fellowship [28]. Data were anonymized and participants provided informed consent prior to each survey, including the peer feedback, and understood that the data would be used for the purposes of evaluation. They also had the opportunity to review and retract the data used in the production of this paper.

## 3. Results

### 3.1. CPhOGH Fellows

All sixteen CPhOGH Fellows responded to both the pre- and post-Fellowship questionnaires with a return of 100% (16/16) response rate. However, not all questions were answered by all. The demographics of the 16 CPhOGH Fellows that participated in the baseline and post-fellowship questionnaires are presented in Supplementary Table S6. The greatest number of respondents were aged 31–40 years (7 respondents) followed by those aged 41–50 years (5 respondents), reflecting the mid-career nature of the majority of the CPhOGH Fellows.

### 3.2. Self-Assessment

#### 3.2.1. Expected Goals of the Fellowship Year

**Figure 1:**
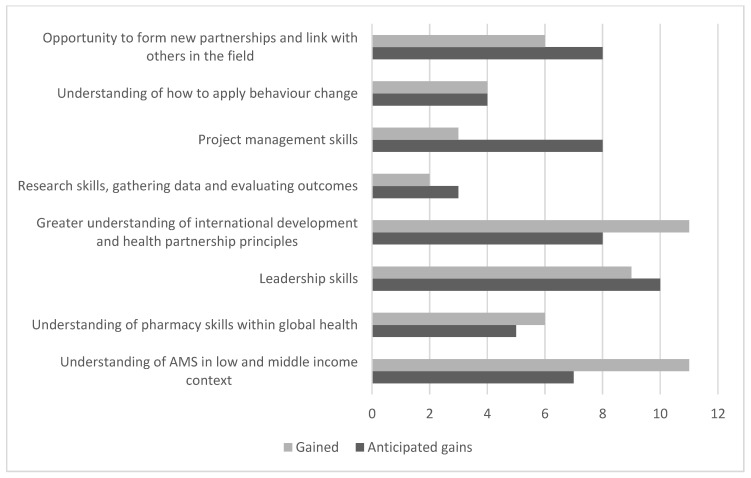
Values represent number of responses to the questions “What do you hope to gain?” and “what have you gained most from the Fellowship year?”.

In the baseline questionnaire, CPhOGH Fellows were asked to select three statements from eight, to reflect what they hoped to gain from the Fellowship year. The responses were then compared with the responses to the post-fellowship questionnaire. Understanding of AMS in a low- and middle-income context and a greater understanding of international development and health partnership principles were both the most popular answers reported after the Fellowship. The responses varied and were different to the outcomes predicted by the CPhOGH Fellows at the start of the Fellowship, as shown in Figure 1. A total of 53 responses were received pre-Fellowship and 52 responses were received post-Fellowship against a request of 48, but one participant qualified this by adding in the comments section that “I could have honestly ticked every box here as I feel that I have been exposed to so many of these opportunities”.

Many CPhOGH Fellows reported that they gained more from the Fellowship than they anticipated:

*“Vastly useful and applicable. I feel I learned more than I taught during my visit! Although I do believe we made a difference for our partnership as well.”*

*“I have gained more skills and knowledge than I anticipated through the Fellowship”.*

*“Will enable me to confidently apply for funding for future projects.”*

#### 3.2.2. Measuring the Outcomes of Volunteering for Education Tool (MOVE-iT)

Summary statistics of Fellows’ feedback on components of the MOVE-iT tool before and post Fellowship are presented in Table 1 (see Supplementary Table S7 for raw data). Overall, there was an increase in the mean and median values of all assessed components and a lower variability in the responses post-Fellowship. The Wilcoxon signed rank test revealed that post-Fellowship, there was a statistically significant increase in Fellows’ perception of their confidence (v = 91, *p* = 0.001), teaching abilities (v = 61, *p* = 0.012), behaviour change (v = 136, *p* = < 0.001), management (v = 66, *p* = 0.003) and communication skills (v = 108, *p* = 0.006). However, the improvement in Fellows’ attitude to work was not statistically significant (v = 92.5, *p* = 0.064).

#### 3.2.3. Leadership Development, Experience, and Knowledge

When baseline and post-Fellowship questionnaires were compared, more leadership and project management activities were reported after the Fellowship compared to before (Table 2). Additionally, new professional development experiences were reported over the Fellowship year (Table 3).

All 16 CPhOGH Fellows agreed that the skills and knowledge they gained during the Fellowship year were useful for the current stage in their careers and were applicable to their positions in the UK. Examples of application of improved leadership skills to everyday work situations were given by several CPhOGH Fellows:

*“I have been able to apply aspects of the leadership skills and knowledge I gained through undertaking the Fellowship to facilitate antimicrobial stewardship work streams which are important to my organisation.”*

*“I have definitely applied the leadership skills learnt through the facilitator study days…. I have also learnt the importance of reflection when things do not always go to plan. I have learnt new ways to deal with conflict which has helped my personal development and ensure that I am able to work effectively.”*

*“I have adapted the flow of work in our team, I am more able to delegate tasks and together we regularly discuss our roles and responsibilities, which has made us more productive as a team.”*

*“I felt the Fellowship has been worthwhile because it introduced me to the leadership course and a tool for me to assess my own leadership skills, identify my gap and act on it.”*

Some CPhOGH Fellows also reported enhanced research involvement and academic teaching opportunities.

*“I have become involved in a research project with health psychologists teaching AMS and behaviour change. I never would have imagined doing this prior to the Fellowship!”*

*“Engagement of the NHS with higher education institutions and improved understanding and participation in research.”*

Additional reflective statements on professional activities and application of skills are shown in Table 4.

A Spearman’s correlation coefficient ρ = 0.23 (*p* = 0.3983) was obtained from the comparison of previous global health experience (Supplementary Table S6) to reflective statements for professional activities and skills undertaken and developed over the CPhOGH Fellowship year (Table 4).

The themes identified from the free text in the post-Fellowship questionnaires were career development, job satisfaction and motivation, communication skills, networking, Global Health and frugal innovation/working with limited resources, education and training (improvements in confidence and adaptation of teaching methods, including the use of behaviour change techniques were reported by the majority of CPhOGH Fellows) and resilience. Table 5 contains the themes that have been identified from the open questions asked in the post-CPhOGH Fellows survey and the corresponding quotes.

### 3.3. Assessment by Senior Colleagues

Results of the leadership questionnaire by colleagues more senior than the CPhOGH Fellows are reported in Table 6. These findings represent the feedback of 26 senior colleagues and responses were received for 14 CPhOGH Fellows by the end of the data collection period; four responses were excluded due to incomplete data sets. One respondent only completed the post-CPhOGH Fellowship section. Two CPhOGH Fellows received feedback from three colleagues; eight CPhOGH Fellows received feedback from two colleagues; and five CPhOGH Fellows received feedback from one senior colleague. Respondents consisted of a wide range of healthcare professionals with six medical consultants, six more senior pharmacy colleagues, five more senior non-pharmacy colleagues, four line managers, three chief pharmacists and two medical colleagues.

There was a shift in responses when the pre- was compared to the post-CPhOGH Fellowship performance for all dimensions. On average, the CPhOGH Fellows progressed from proficient/established score of 2.5 pre-CPhOGH Fellowship to strong/excellent score of 3.3 post-CPhOGH Fellowship. Only one CPhOGH Fellow was considered to have the same overall score (2.9) for the pre-and post-Fellowship questionnaire. This performance was rated proficient/strong overall, across all dimensions. The respondent did not provide any narrative for this individual (see supplementary Figure S1).

### 3.4. Impact of the CPhOGH Fellowship on the Performance of Participating Pharmacists from a Senior Perspective

The one theme that constantly featured among all the dimensions was confidence. This was often regarded as a result of a change in the perception of self-efficacy, referring to an individual’s belief in their capabilities to perform a task. The respondents felt that the Fellowship had empowered the CPhOGH Fellows to be more confident in striving for things that the CPhOGH Fellows perceived outside their reach. These included confidence to voice their opinions, integrating outside their comfort zone, accepting new challenges, and volunteering to lead a working group or project. The following feedback reflects this:

*“Professionally the Fellow is more confident, able to allow their voice to be heard. The Fellow has developed their ability to manage change and people skills and works confidently within wider teams.”*

*“Professionally the Fellow is more confident, more strategic in their thoughts and planning, ambitious and excited for improvement. The Fellow has developed their networks extensively, working across networks globally with ease.”*

*“Increased confidence in ability to adapt to new situations and different environments. Ability to manage stressful situations and turn them to an advantage.”*

*“Confidence, ability to think outside the box and use successful techniques in a different context.”*

A total of 96% (*n* = 25) of the seniors thought the CPhOGH Fellows were ready for more senior roles and 88% (*n* = 23) of them would recommend the CPhOGH Fellowship to others pharmacy colleagues.

*“It has been a pleasure to watch the Fellow grow during the year and overall, this programme has been a positive influence on the department as a whole.”*

### 3.5. Contributions, Achievements and Communications

Feedback from the CPhOGH Fellows on the scope of the work published/presented was wide ranging, with national and international representation at conferences, webinars, blogs, podcasts and community engagement events (see Supplementary Tables S4 and S5).

## 4. Discussion

This evaluation aimed to consider the impact, if any, of participation in the CPhOGH Fellowship, particularly in relation to leadership skills. It also deliberates whether this programme offers a development opportunity to provide demonstrable benefits to pharmacists as individuals and the wider NHS. The overall impression from the responses provided in the questionnaires indicates that all CPhOGH Fellows underwent a great deal of personal and professional development over the one-year Global Health Fellowship.

The responses indicate the significance of the Fellowship in honing vital traits beneficial to individual pharmacists and the NHS. Research in the UK has highlighted that the best performing hospitals were those in which staff demonstrated high levels of engagement in decision making and where there was evidence of distributed leadership in the organisation [29]. Increasing the confidence and leadership skills of pharmacists not employed in traditional leadership roles provides additional benefit to their organisations (evidenced by 10 fellows managing new aspects of service since commencing on the fellowship) and the NHS. This aligns with participants’ most anticipated gain from the Fellowship from the pre-Fellowship survey—development of leadership skills. Although the impact of the Fellowship on pharmacists’ ‘Attitude to work’ was not statistically significant, descriptive statistics show an increase in the mean and median values of respondents’ perceptions of their abilities after the Fellowship. Improvements in confidence were mirrored in the feedback from senior colleagues when asked to assess the Fellow’s performance. The use of feedback from senior colleagues is similar to the recommendation to use 360° assessments to move beyond the weaknesses of self-reported changes. [29,30,31].

The majority of CPhOGH Fellows reported that they gained the most from understanding AMS in an LMIC context, understanding international development and health partnership principles and leadership skills. This supports the intention of CwPAMS—to strengthen AMS capacity in LMIC health institutions as part of collaborative, partnership efforts, whilst providing leadership training as part of the CPhOGH Fellowship. In retrospect, this question was too restrictive as it allowed only three options to be selected from a pre-set of eight. There were more responses than requested and there was a spread of responses for all statements. The narrative provided allowed additional and more extensive insights to be captured.

Nearly a third of the CPhOGH Fellows had previous experience in global health. The results indicate insufficient evidence of a correlation between Fellows’ previous global health experience and reflective statements for professional activities and skills undertaken and developed over the CPhOGH Fellowship year. Hence, factors other than previous global health experience could be responsible for the professional activities and skills undertaken and developed during the Fellowship year.

In some instances, it was difficult to unpick the developments and progression of the CPhOGH Fellowship experience from their routine job roles, as many CPhOGH Fellows reported that they practiced many of the professional activities and utilised the leadership skills regularly. Despite 74% of CPhOGH Fellows having 11 or more years’ experience as a pharmacist, undertaking some of the professional activities routinely, most Fellows reported improvements or changes to the way in which they undertake the activities they were asked to self-reflect on. For example, CPhOGH Fellows reported more structured knowledge of tools and application of these in practice, resulting in a more holistic approach when undertaking quality improvement and project management tasks.

This fellowship has facilitated increased opportunities and new experiences for the Fellows with extended networks and visibility on a national and international level. This is evidenced by feedback from senior colleagues who credited the positive influence that the CPhOGH Fellows exhibited within their own departments to motivate others and act as role models. Workplace-based leadership training has been shown to increase willingness to lead [32]. These qualities and skills were challenged during the COVID-19 pandemic and it was acknowledged that the CPhOGH Fellows responded positively and were able to adapt to new ways of working.

The follow-up questionnaire revealed that job satisfaction and motivation improved, with seven CPhOGH Fellows reporting a change in job role and five receiving a promotion. This is similar to reports from MacPhail et al. (2015), where ‘The clinical leadership programme significantly increased willingness to take on leadership roles’ (93%), and participants reported that they were more willing to take on a leadership role within their team [32]. Progress was noted across all domains by senior colleagues when considering the NHS Healthcare Leadership Model and the RPS peer assessment tool.

Despite a small initial cohort of participants, the evaluation and feedback indicate that the CPhOGH Fellowship is beneficial as a development opportunity for pharmacists. However, future programmes should be offered to a wider number of pharmacy staff, including technicians, to allow for more representative analysis. Feedback should also be sought from LMIC partners to facilitate a more holistic review of performance and support bidirectional learning.

## 5. Limitations

The benefits of the CPhOGH Fellowship are intertwined with the benefits of participating in a global health project and therefore, it is difficult to identify the independent benefits of each. In hindsight, the leadership skills questionnaire for senior colleagues should have been completed at baseline and after the Fellowship to reflect answers more accurately. In comparison with other global health Fellowships [8], the number of participants included was small and the time spent in the partnership country was limited. Tools that elicit self-reported attainment and behaviour changes are considered to provide weak evaluation evidence, are of variable accuracy [33] and most studies use unvalidated tools. In this study, however, we combined a validated tool (MOVE-iT) for self-reporting [25] pre- and post-fellowship, [34] alongside independent peer assessment using nationally recognised leadership framework tools [18,28]. The use of a non-random population could also impact the results of our findings. Hence, the inference from this study should be treated with caution. Whilst this paper focusses on the impact of a Global Health Fellowship on UK-based pharmacists, it does not account for the leadership skills and development of pharmacists from LMICs involved in the CwPAMS projects.

Due to the impact of the COVID-19 pandemic, there was limited opportunity to obtain feedback from the Fellows’ LMIC partners on the progress of the Fellows during the CwPAMS project.

## 6. Conclusions

This was the first global health fellowship for pharmacists in the UK. Overall, the Fellowship was a valuable experience for all those that took part in it. The engagement in the questionnaires and the extensive narrative provided by the Fellows showed their commitment to the Fellowship and the many outputs derived from it. The analysis of the data provided suggests that this CPhOGH Fellowship led to the upskilling of more confident, motivated pharmacist leaders with a passion for global health. This supports the NHS’s long-term plan “to strengthen and support good compassionate and diverse leadership at all levels” [4]. There was some constructive feedback for how the Fellowship could be improved in anticipation of the offer of another CPhOGH Fellowship, as benefits can clearly be seen by CPhOGH Fellows and senior colleagues alike.

## Figures and Tables

**Table 1:** Summary statistics for cumulative Likert-rated responses for the components of MOVE-iT.

	Pre-Fellowship			Post-Fellowship			*p*-Values
	Mean	Median	Standard Deviation	Mean	Median	Standard Deviation	
Confidence	53.31	52	6.23	58.69	61	4.81	0.001
Teaching	17.31	18.5	2.75	19.13	19	1.89	0.012
Behaviour change	19.31	19	5.50	22.88	24.5	5.56	<0.001
Management	17.50	18	3.39	19.81	21	1.83	0.003
Attitude to work	35.81	36	3.56	38.00	37	3.41	0.064
Difficult communication	15.00	15	4.07	18.44	18.5	2.99	0.006

**Table 2:** Leadership and project management activities experienced by the CPhOGH Fellows pre- and post-CPhOGH Fellowship programme.

Activities	Pre CPhOGH (*n* = 15)		Post CPhOGH (*n* = 16)			
	Yes	No	Yes (through Fellowship)	Yes (Alternative Route)	No	Not Answered
360 Assessment Questionnaire	4	11	7	3	6	
NHS Healthcare Leadership Model Self-Assessment	2	13	15	1		
Other leadership self-assessment	5	10	7	1	5	3
Project Management Course	6	9	10		6	
Myers–Briggs Type Indicator questionnaire	8	7	15			1
Other personality type indicator questionnaire	5	10	2	3	11	
A formal/semi-formal discussion with a mentor for your professional activities	5	10	16			
Written a project plan	10	5	15	1		
Formally led a project or project deliverable	11	4	15	1		
Led a quality improvement project	10	5	7	4	5	

**Table 3:** Professional activities undertaken by CPhOGH Fellows during the Fellowship year, including new experiences.

Activities Undertaken for the Last 12 Months (June 2019–June 2020)	*N* = 16 (New Experience)
Publication/presentation of work at a conference	12 (3)
Publication of work for a journal	9 (3)
Publication/presentation of work within Trust setting	13 (3)
Promotion of work on social media	11 (6)
Collaboration on work within Trust	13 (2)
Collaboration on work at a regional/local level (outside Trust)	12 (3)
Collaboration on work at a national level	13 (7)
Collaboration on work at an international level	13 (7)
Utilised a mentor	15 (8)
Become a mentor	6 (4)
Changed job role	7
Had a promotion	5
Undertaken Faculty assessment	4 (1)
Written a business case	7 (1)
Undertaken an audit	14
Undertaken a project with a focus on quality improvement	12 (1)
Undertaken teaching	16
Enrolled or undertaken a leadership course	14 (9)

**Table 4:** Reflective statements for professional activities and skills undertaken and developed over the CPhOGH Fellowship year.

Reflecting between June 2019–June 2020 State How Much You Agree with the Following Statement (*n* = 16)	Strongly Agree/Agree	Somewhat Agree	Neutral	SomewhatDisagree	Strongly Disagree/Disagree
I undertake more MDT work compared to a year ago.	7	2	4	1	2
I am more likely to work with other disciplines on a regular basis	8	3	3		2
I have worked across disciplines in delivering AMS/IPC	14		1		1
I find myself working more with different professional groups	11	2	1		2
I am more confident to approach people I have never worked with before compared to a year ago	14		1		1
I am happy to work with/approach people who work outside the NHS in order to collaborate compared to a year ago.	14	1			1
I have started managing a new aspect of service.	10	2	2		2
I recognise the need to review/manage /introduce a new aspect of service.	12	2	1		1
I have become more involved in research.	7	3	2	1	3
I have made changes to the way in which I work as a team	12	3	1		
I have made changes to the way in which I engage with others in the work environment.	13	3			
I have made changes to the way in which I teach	13	2			1
I have made changes to the way in which I engage with the wider Trust/ organisation.	10	1	2		3
I have made changes to the way I practice.	13	1	1		1
I have made changes to the way in which I engage with others outside a work environment.	12	2	1		1

**Table 5:** Themes from Open questions in the post-CPhoGH Fellows survey.

Themes from Open Questions	*Selected Quotes*
**Career development** In addition to the seven CPhOGH Fellows who reported a change in job role and the five CPhOGH Fellows who reported a promotion (Table 3), the written feedback supports improved development and professional progression through the experiences of the Fellowship programme.	*“It has also given me the confidence to apply for a managerial role, I wouldn’t have considered only months before.”* *“I have had opportunities e.g. projects with significant budgets which I believe I may not have been offered if it weren’t for the Fellowship”* *“The Fellowship has empowered me to push myself further and broaden my horizons.”* *“It has provided me with the tools for the next stage of my pharmacy career.”* *“Lots of experiences during the last year motivated me to change role. Fellowship was one of them.”*
**Job satisfaction/motivation** The results of the MOVE-iT tool, shown in Table 1, do not indicate a significant change in attitude to work. However, the following statements suggest improved job satisfaction, pro-active working and motivation.	*“Improvements in staff happiness and retention.”* *“Increased confidence. Increased motivation”* *“The Fellowship has given me another focus and has improved my motivation and job satisfaction in my role.”* *“I’ve expanded my goals and through the support of my peers and Fellows I have achieved things I would never have dreamed of 18 months ago.”* *“The CwPAMs projects and CPhOGH Fellowship has offered unique opportunities for us which have been very enjoyable. They have encouraged me to work hard and with more enthusiasm than before and I feel very happy and proud to work within the pharmacy profession.”*
**Communication skills** Improvements in managing challenging conversations and difficult people were reported in the post-CPhOGH Fellows MOVE-iT survey when compared to the baseline (Table 1). This is further substantiated by the following:	*“My improved negotiation and communication skills, especially in tricky circumstances”* *“The coaching on the Fellowship weekend really allowed me to explore this in a safe environment and practice difficult conversations”* *“Coaching course delivered on the Fellowship weekend. This was amazing and has really improved my confidence and communication style.”* *“I have learnt new ways to deal with conflict which has helped my personal development and ensure that I am able to work effectively.”*
**Networking** Collaboration within Trusts, at a regional, national and international level, was reported by the majority of CPhOGH Fellows (Table 3). Stronger and more cohesive networks were developed locally as illustrated. Collaborations across sectors and specialties and outside the NHS were also fostered as described in Table 4 and by these CPhOGH Fellows:	*“The networks I have made also were useful in other areas of healthcare. Such as project plans for in house service development.”* *“Wider network which I can call on to enhance what we are doing at work.”* *“felt more empowered to work differently and innovatively within and outside our organisation.”* *“It has connected me to inspiring and like-minded colleagues who all have a passion for improving global health in particular focusing on AMR.”* *“I feel more confident to network now and have learnt a huge amount from positive role models outside of healthcare.”* *“Greater collaboration. I have paired up the Uganda researchers with colleagues in South Africa to write a grant proposal.”* *“Improved ability of staff involved to work beyond the traditional boundaries and to consider how collaborative efforts can provide further benefits to all.”*
**Global Health and Frugal Innovation/Working with limited resources** The CPhOGH Fellows demonstrated a greater ability to work within the confines of limited resources as indicated by the greater agreement with the statements in the MOVE-iT (Table 1). Examples of benefits of this in the UK setting are illustrated here:	*“In addition, having increased understanding of the global health context has helped me believe that anything is achievable with the resources in the UK, you just need the will to make change.”* *“Ability to produce outcomes and results in a very resource limited setting”* *“Greater awareness of how LMIC institutions are run and how cohesive relations are built. This is something that can benefit NHS organisations”* *“More appreciation of what the NHS has to offer (as a patient and an employee) and how improvements do not always require money and more often, a better understanding of how things actually work in practice.”* *“The opportunity to participate in the CwPAMs project and the CPhOGH Fellowship has been eye opening. I have learnt how to improvise and find solution despite limited resources. I also learnt to be more adaptive to the environment and change way of working depending on the local context. This has been very useful given the COVID-19 pandemic.”* *“It has been worthwhile, as it has given me greater exposure to the way in which a pharmacists role can be done even with great lack of resource. It has shown me how when undertaking the role of a leader you are able to accomplish a lot, despite any challenges.”*
**Education and training** All CPhOGH Fellows considered education and training to be part of their job roles prior to the Fellowship and CwPAMS experiences (Table 3). However, improvements in confidence and adaptation of teaching methods, including the use of behaviour change techniques, were reported by the majority of CPhOGH Fellows (Table 1 and Table 4). Delivery and provision of education and training was also reported to be further reaching. This is highlighted here:	*“The key benefits to my trust is the Fellowship has changed the way we conduct education and training through the introduction of behaviour change.”* *“…. link with partnership and UK local university school of pharmacy to support overseas opportunities for undergraduate programme”* *“Far greater understanding of requirements to deliver high quality education at scale”* *“Together with colleagues in Uganda we developed a MOOC that has been accessed in 50 countries by over 2000 learners on AMS.”* *“education strategies have changed to move away from didactic teaching to more workshop type training and in addition our work has made the team more visible in the trust. It also has embedded AMS into the long-term strategy of all areas.”*
**Resilience** When the CPhOGH Fellows were asked how they believed they had coped with the COVID-19 pandemic in April and May 2020, five answered extremely well, seven answered very well and three answered somewhat well. One CPhOGH Fellow did not respond. For some CPhOGH Fellows, they reported that they used their experiences of working in new and uncertain areas during their CwPAMS placements to be helpful:	*“ a better sense of self-awareness (after a lot of reading/research into this area, borne from the Fellowship study days)”* *“I built upon my experience in my new role and also the increased leadership skills I have been building on during the Fellowship which helped me to cope when I was redeployed to a clinical role during COVID.”* Others considered perspective of the situation to be important and utilised the leadership and educational skills they had developed: *“I think the experiences of the past year have helped in dealing with the COVID19 emergency response and this has put into context what is important in life.”* *“Positive outlook. Good communication. Open teamwork. Task delegation. Feeling prepared by having additional teaching.”* *“I was re-deployed to support a clinical research team and having international experience…during the Fellowship and working with and managing international teams helped me cope with being in a new environment and responding to new and as yet unknown research needs and questions.”* *“Leadership demonstrated by caring for my colleagues and my team’s mental health and wellbeing. I take the time to listen and be there for colleagues, despite my very busy schedule.”*

**Table 6:** Leadership questionnaire adapted from RPS Peer Assessment Tool and Healthcare Leadership Model.

Statement	Pre CPhOGH (*n* = 25)					Post CPhOGH (*n* = 26)				
	Essential	Proficient	Strong	Exceptional	U/C*	Essential	Proficient	Strong	Exceptional	U/C*
**Teamwork**										
The pharmacist listens attentively to other team members and values their suggestions		3	17	5			1	10	15	
The pharmacist is an established and effective member of a multidisciplinary team	2	4	15	4			1	10	15	
The pharmacist is consulted for advice which requires their in-depth professional expertise	1	6	12	6				10	16	
The pharmacist consistently works effectively across boundaries to build relationships and share information, plans and resources.	2	6	9	7	1		1	5	19	1
U/C* Unable to comment										
**Supporting quotes** *“…. ability to network and be collegiate widens the scope and impact of Fellow work, and the Fellow is fundamental in keeping our team connected and on task.”* *“Their positive, "can-do" attitude and unrelenting support for ALL members of the team means you can face any challenge with them!”* *“The Fellow is a core member of and contributor to the Antimicrobial Stewardship Committee…. developed strong working relationships with clinicians, pharmacists and specialist nurses in both hospital and community settings…. taken an active leadership role in the Trust response to COVID-19 working across site and specialities.”* *“The Fellow has always been strong team player and has developed strong networks…sought for expert clinical advice at a National level”*										
**Influencing for Results**										
The pharmacist is able to share issues and information to help others understand his/her point of view	1	11	8	5			3	11	12	
The pharmacist tailors his/her communication group or presentation according to the audience or stakeholder.	4	8	9	3	1		2	14	10	
The pharmacist directly or indirectly links different working groups across the organisation to achieve a common goal.	3	7	9	5	1		6	9	11	
The pharmacist gains reputational influence by sharing experience and best practice nationally	3	5	10	4	3		5	6	14	1
U/C* Unable to comment										
**Supporting quotes** *“The Fellow is able to communicate and build relationships with anyone, irrespective of position or background. They have been proactive in sharing theirs and the team’s work both locally and at conferences.”* *“The Fellow has worked hard to establish their place in the team with confidence, they now have a much stronger voice in our QI huddles and across the department”* *“As Lead Antimicrobial Pharmacist the Fellow has to communicate with a variety of professionals in a number of different settings, all of whom may have differing agendas e.g. harmonisation of antimicrobial guidelines following the merger of two large hospital Trusts….has spoken at national conferences and set up a professional Twitter account for dissemination and sharing of information and networking.”*										
**Vision, Motivation and Developing Capability**										
The pharmacist creates a clear vision of the future for his/ her team in accordance with the organisations vision.	3	12	7	3		1	3	11	11	
The pharmacist often looks for opportunities to develop him/herself and learn things outside his/her comfort zone.	2	11	9	3			3	9	14	
The pharmacist often provides constructive feedback to his/her team to help them focus on the right area in order to develop professionally	4	6	6	3	6	1	6	8	6	5
The pharmacist is involved in strategy planning	4	7	9	2	3	2	4	11	8	1
U/C* Unable to comment										
**Supporting quotes** *“The Fellow sees the bigger picture but is also able to hone in on the key steps required to implement change and is detail-orientated when it is appropriate.”* *“very sensible and objective in discussions, also very good at providing feedback, identifying colleagues who need support and providing help to the best of their abilities.”* *“The Fellow has taken on the responsibility of being a diploma tutor…….and also actively engages with other diploma students and gives constructive and positive development suggestions.”* *“Lead role in development of Antimicrobial Stewardship strategy…. involvement in Trust Infection Service strategy working group”* *“The Fellow has been pivotal in planning the next steps of the project and disseminating the links work at a national level”*										
**Inspiring shared purpose**										
The pharmacist demonstrates the characteristics of a role model of the Pharmacy profession.	1	4	12	6	2			9	16	1
The pharmacist inspires others, even when they are under pressure, by helping them to focus on the value of their contribution	1	10	8	6			3	10	13	
The pharmacist supports his/her colleagues to keep challenging others cohesively to achieve a shared purpose.	1	10	9	5			3	12	11	
U/C* Unable to comment										
**Supporting quotes** *“The Fellow is supportive, encouraging and inspiring in their enthusiasm, drive and outlook.”* *“The Fellow responded positively to the COVID crisis, showing calm yet strong leadership for the staff delivering clinical services to ward areas.”* *“The Fellow has managed the recent changes in working due to COVID-19 with grace and understanding…been a role model to their colleagues. Fellow has forged strong links with psychology colleagues at the university to provide behaviour change education to Stewardship Committee members and regularly demonstrates what they has learned from these sessions in leadership role.”*										
**Managing Change**										
The pharmacist is able to adapt to different way of working especially in times of crisis	1	8	11	3	2			10	15	1
The pharmacist consistently reflects on his/ her service and manages processes of change or service improvement	4	4	11	3	3		1	16	7	2
The pharmacist applies a behaviour change approach in change management or education and training	3	12	2	3	5		3	12	8	3
U/C* Unable to comment										
**Supporting quotes** *“The Fellow is very good at identifying the context for change and making on-going assessment of the potential impact of change.”* *“The Fellow has embraced new concepts of behaviour change in terms of education and training and has applied this new learning into practice.”* *“The Fellow has worked hard to develop their confidence and her voice in the team, now confidently able to establish their ideas into change.”* *“Use of QI methodology to understand problems, adapt processes and work differently.”* *“…. can take on a variety of challenges, whether that involves service improvement, additional clinical responsibilities or education and training.”* *“Working in a LMIC has provided an opportunity to apply skills in a new context and learning can be applied in future roles.”*										
**Innovative working and practice**										
The pharmacist strives to improve his/her service within the limitation of resources.	3	8	9	3	2		2	16	8	
The pharmacist is able to analyse essential data and utilise the results to improve services.	2	8	8	4	3		3	13	8	2
The pharmacist is able to identify the key stakeholders and understand their agenda.	4	12	4	5			4	12	10	
The applicant takes the lead to ensure innovation produces demonstrable improvement	2	11	6	2	4	1	3	13	8	1
The Fellow effectively strives to improve quality within limitations of service	3	9	7	5	1		3	13	10	
The pharmacist recognises and implements innovation from the external environment.	3	12	4	3	3		3	13	8	2
U/C* Unable to comment										
**Supporting quotes** *“The Fellow has an open mind to new ideas/innovation and change, i.e., not afraid of trying new ways of working. The Fellow has an increased awareness of stakeholder engagement and project work and in mindful of influences.”* *“Pushed forward with new prescribing models to address deficiencies brought about by staffing issues”* *“Recognises areas for improvement using resources such as audit and incident reporting, makes an action plan and delivers on these plans”* *“…interested in research and finding innovative ways of doing things…also good at giving due consideration to new and innovative ideas, as observed during our partnership project. I was impressed by… enthusiasm and dedication”*										

## Data Availability

Data is contained within the article or supplementary material.

## Supplementary Materials

The following are available at https://www.mdpi.com/article/10.3390/healthcare9070890/s1, Table S1: Self-assessment questionnaire (baseline), Table S2: Post-CPhOGH Fellowship Self-assessment questionnaire, Table S3: Assessment of CPhOGH Fellows Leadership skills by Senior Leaders, Table S4: Additional activities of the CPhOGH Fellows during the Fellowship year Table S5: Themes from the additional activities of the CPhOGH Fellows, Table S6: Demographic of the CPhOGH Fellows Table S7: Statements for the components of MOVE-iT, Figure S1: Average Leadership Dimension Score per CPhOGH Fellows.
