# Supplementary material for: Assessing the Impact of a Global Health Fellowship on Pharmacists’ Leadership Skills and Consideration of Benefits to the National Health Service (NHS) in the United Kingdom
Source: Healthcare (Basel). 2021 Jul 15;9(7):890. doi: 10.3390/healthcare9070890 (PMC8304379; doi:10.3390/healthcare9070890)
Supplement: Supplementary file 1 [file healthcare-09-00890-s001.zip › healthcare-1260156-supplementary.pdf]

Supplementary Material

Table S1. Self-assessment questionnaire (baseline).

Question	Included / excluded	Rationale
1. What do you hope to gain most from the fellowship year?<br>(Select maximum 3; you can provide more context or information by selecting other)<br>• Understanding of AMS in low- and middle-income context<br>• Understanding of pharmacy skills within global health<br>• Leadership skills<br>• Greater understanding of international development and health partnership principles<br>• Research skills, gathering data and evaluating outcomes<br>• Project management skills<br>• Understanding of how to apply behaviour change<br>• Opportunity to form new partnerships and link with others in the field<br>If you wish to add more information, you can use this comment box.	Excluded	Question to determine what has been perceived to be gained from the fellowship used from post-fellowship questionnaire
2. Do you currently have a dedicated/allocated lead/formal role in delivering any of the identified objectives or monitoring and learning indicators for CwPAMS?<br>Yes/No	Excluded	Question repeated in the post-fellowship questionnaire
3. Which indicator or activity do you have the lead for delivering?	Excluded	Question repeated in the post-fellowship questionnaire
4. Within the last 12 months (i.e. 1st June 2018 to 31st May 2019) have you completed the following:<br>• NHS healthcare leadership model self-assessment<br>• Any other leadership self-assessment<br>• A project management course (this can be in the last year<br>• Myers–Briggs Type Indicator Questionnaire<br>• Any other personality type indicator questionnaire<br>• Had a formal/semi-formal discussion (ie a pre-booked discussion not adhoc) with a mentor for your professional activities<br>• Written a project plan<br>• Formally led a project or project deliverable (ie. you were responsible for delivering a project and reporting the findings formally)<br>• Led a quality improvement project	Excluded	Question repeated in post-fellowship questionnaire to include before June 2019 (inception of Fellowship) and since June 2019. Additional question added to include 360° feedback

Use this section to provide clarification or further information on any of the above		
5. Do you currently use social media for professional activities?<br>Yes, fully<br>Yes, somewhat<br>Not at all	Excluded	Not the only form of communication. Does not directly reflect leadership.<br>Personal preferences.
6. Please state how much you agree with the following items (Likert rated):<br>**Confidence**<br>• I am confident in my ability to manage myself in a clinical environment.<br>• I am confident in my abilities to work independently when necessary.<br>• I am confident in my ability to deal with the unexpected.<br>• I am confident in my ability to be adaptable and innovative as a leader.<br>• I am confident in my ability to adapt and be flexible clinically.<br>• I am confident in my ability to adapt and be flexible in general.<br>• I am confident in my ability to find solutions despite limited resources.<br>• I am confident in my ability to apply clinical skills to another context.<br>• I am confident in my work.<br>**Life satisfaction**<br>• In most ways my life is close to my ideal.<br>• The conditions of my life are excellent.<br>• I am satisfied with my life.<br>• So far I have gotten the important things I want in life.<br>• If I could live my life over, I would change almost nothin<br>• Taking everything into consideration, I am satisfied with my job.<br>**Teaching**<br>• I am confident in my ability to teach others	Included confidence and teaching from the MOVEIT tool questions but excluded life satisfaction questions from analysis	Participants questioned why life satisfaction questions were asked in the post-fellowship questionnaire as they did not seem relevant during a pandemic. and could not be compared to baseline.<br>Teaching question excluded as skills do not reflect / linked to leadership.
7. In the last month…..<br>**Cultural**<br>• I demonstrated a good awareness about how culture influences health.<br>• I frequently demonstrated cultural sensitivity.<br>• I was constantly conscious of culture when working with patient<br>**Adapting communication**	Excluded cultural and adapting communication<br>Included teaching, behaviour change, management, attitude to work and difficult communications	Not all participants were in LMIC placements or exposed to experiences to be able to answer these questions. Participants did not find these questions relevant

- I changed the way I speak so that somebody can understand me (e.g. purposely spoke slower and clearer).
- I changed the way I communicate to make it more contextually appropriate (e.g., to make it more culturally appropriate).
- I frequently relied on my non-verbal communication (e.g. hand gestures).

Teaching

- I demonstrated I'm a good teacher.
- I adapted the way I teach to make it better for the learner.

Difficult communications

- I demonstrated that I am skilled in challenging conversations, even in high pressure situations.
- I demonstrated that I am able to manage difficult people effectively.
- I frequently dealt with difficult people.

Behaviour change

- I am able to empower patients to help themselves.
- I am able to empower colleagues to help themselves.
- In my work I have demonstrated skills in changing colleagues' behaviour.
- In my work I have demonstrated skills in encouraging and supporting patients to change behaviour.

Management

- I allocated tasks.
- I co-ordinated colleagues.
- I demonstrated I am able to plan and organise.

Attitude to work

- I was frequently proactive at work (e.g. used my initiative, got on with things, thought on my feet).
- I demonstrated that I am able to cope in work (e.g. able to deal with stress).
- I demonstrated that I am particularly good at working as part of team.
- I demonstrated I'm good at dealing with the unexpected
- I frequently had to find solutions despite limited resource
- I demonstrated I am able to find solutions despite limited resources.

Participant ID details. We need to be able to link your answers on this survey with answers on surveys in the future or past. Please share your email address so we can do that. If you do not want to share your email address for this purpose, please generate a code by entering in the following this order: The 3rd letter of your first name*the 4th letter of your first school*the first letter of your mother's first name*the date that you were born i.e., the 'day of the month from 01 to 31*the second letter of your last name. This should be a 6 figure string of letters and numbers that allows us to anonymously identify you

8. Gender (M/F	Excluded	Repeated in post-fellowship questionnaire. Demographics included from this.
9. Age (Freetext)	Excluded	Repeated in post-fellowship questionnaire. Demographics included from this.
10. Job title		
11. Employment status (before trip)	Excluded	Irrelevant
12. In what sector is your primary role (i.e > 50%)	Excluded	Repeated in post-fellowship questionnaire. Demographics included from this.
13. Years since registration	Excluded	Repeated in post-fellowship questionnaire. Demographics included from this.
14. Nationality	Excluded	Irrelevant
15. Name of partnership	Excluded	Irrelevant
16. Is this your first international placement? (please provide additional information)	Excluded	Repeated in post-fellowship questionnaire. Demographics included from this.
17. Are you using any annual leave for the trip?	Excluded	Irrelevant for purposes of this paper
18. What is your primary motivation for the trip?	Excluded	Irrelevant for purposes of this paper
19. Do the following support your trip?<br><br>Colleagues<br>Deanery<br>Line Manager<br>Trust	Excluded	Irrelevant for purposes of this paper
20. Additional Pre-Placement Questions<br><br>• I would feel comfortable working outside of my competence<br>• I would feel comfortable working in a high-risk situation<br>• I think I will make a major difference to the host country within my trip<br>• I have an excellent understanding of my current professional knowledge e.g. I understand how much I currently know and what I need to work on in the future	Excluded	Irrelevant for purposes of this paper
21. Do you consent to the following?		
22. At the end of the fellowship, I will feel the fellowship has been worthwhile IF.....	Excluded	Irrelevant for purposes of this paper. This paper focuses on changes achieved / perceived

23.	Have you had an in-country visit already?	Excluded	Irrelevant for purposes of this paper
24.	About the LMIC placement<br>• Length of stay<br>• Country visited<br>• Town/City visited<br>• Type of facility/placement (e.g. rural hospital, regional referral hospital, community work)	Excluded	Repeated in post fellow questionnaire. Not all participants knew the details at the point of the survey.
25.	Thinking about your most recent international placement, please state how much you agree with the following statements:<br>• I felt engaged with the project throughout<br>• I learnt the host language<br>• I felt my skills were best utilised e.g. my skills were effectively utilised in the host country<br>• I interacted with more patients each day than I would in the UK<br>• I experienced a greater variety of conditions than I would in the UK<br>• I copied the behaviours of the staff in the host country e.g. agreed with and internalised lots of the knowledge, attitudes, skills and behaviours of other staff in the host facility<br>• At least once, I was aware of my opinions or perspectives changing in a significant way<br>• It was easy to accommodate the experiences I had into my own view of reality<br>• I understood the local context e.g. culture, customs, hierarchies, power dynamics	Excluded	Irrelevant for the purposes of this paper
26.	Will you be going on a second visit to the LMIC as part of CwPAMS	Excluded	Total duration of in-country time captured in post- fellow questionnaire and included in demographics
27.	Which of the following aspects of your placement were similar to the UK? (Please check all applicable)<br>• Licencing, protocols and regulations<br>• Health and safety<br>• Host country Culture<br>• Healthcare professional ethics (e.g. acting ethically)<br>• None of the above	Excluded	Irrelevant for the purposes of this paper
28.	What opportunities were available to you during your placement?<br>• To lead and have responsibility<br>• To visit more than one health facility<br>• To explore life outside of the hospital and immerse yourself in local culture<br>• None of the above	Excluded	Not all participants had undertaken an in-country placement at the point of this survey. Incomplete data capture.

29. The skills and knowledge I gained during my placement.... (Please check all applicable)<br><br>...are useful at the current stage in my career .<br>...are applicable to my UK position<br>None of the above	Excluded	Repeated in the post-fellow's questionnaire. Not all participants had undertaken an in-country placement at the point of this survey. Incomplete data capture.
30. Which of the following are correct about the local staff you met on your placement? (Please check all applicable)<br>• They were under time pressures<br>• Many left or moved facilities within my stay<br>• I felt encouraged by them<br>• I had a local role model I experienced communication difficulties<br>• I engaged with them frequently<br>• They had adequate financial and human resources<br>• They were critical of volunteers and the project<br>• Hospital leaders were engaged with the project<br>• I engaged frequently with local staff<br>• I have stayed in touch with many of them<br>• There was frequently a more clinically knowledgeable person working alongside me<br>• There was frequently a more knowledgeable person (about local culture) working alongside me<br>• Many have adopted some of my skills, knowledge and attitudes and used this in their practice<br>• It was obvious that we had many shared values<br>• None of the above	Excluded	Irrelevant for the purposes of this paper
31. Did you experience any of the following as a result of your placement? (Please check all applicable)<br>• Health consequences (injuries, illness etc.)<br>• Loss of earnings (for time away)<br>• Loss of pension or other employee benefits<br>• Exposure to corruption<br>• None of the above	Excluded	Irrelevant for the purposes of this paper
32. I felt the work on the placement was...<br><br>• Too easy, repetitive or boring<br>• Challenging but achievable	Excluded	Repeated in post-fellow's questionnaire

• Overwhelming, beyond my capacity and frustrating<br>• None of the above		
33. What support did you have access to? Please check all applicable<br>• UK Mentor<br>• Mentor in LMIC<br>• Supervision/support from other volunteers in LMIC (i.e. linking of junior and senior volunteers)<br>• Supervision/Support from local staff in LMIC<br>• Support from other CwPAMS volunteers<br>• Formal support structure in LMIC (e.g. access to HR)<br>• Support from volunteers working on another project (in country)<br>• Frequent feedback from a local senior colleague<br>• Frequent feedback from a western senior colleague<br>• Support from CPA or THET<br>None of the above	Excluded	Repeated in post-fellow's questionnaire
34. On average, how much in total did you spend on the placement? (Including flights, accommodation, project fees, living expenses, vaccinations etc.)	Excluded	Irrelevent
35. How many other projects were working in the healthcare facility?	Excluded	Participants were not always aware of the answer to this question having limited time in country.
36. I critically reflected upon my experience (Please check all applicable)<br>• During my placement<br>• Upon return from my placement<br>• Formally<br>• Informally<br>• None of the above	Excluded	Repeated in post Fellow's questionnaire
37. Generally I felt the experience was...	Excluded	Repeated in post-fellow's questionnaire for complete data capture.
38. Do you have any comments regarding the questions in this section?	Excluded	No additional feedback
39. Thank you very much for completing the questionnaire. Do feel free to use this section to provide additional comments	Excluded	No additional feedback

Table S2. Post-CPhOGH Fellowship Self-assessment questionnaire.

The questionnaire is an important part of the CPhOGH Fellowship and CPA's service provision. By proceeding to the next page:

I consent to CPA collecting and using the information about me that I provide for the purposes of the survey

I have read, understand and agree to the information provided above

Question	Included / excluded	Rationale
1. What do you hope to gain most from the fellowship year?<br>(Select maximum 3; you can provide more context or information by selecting other)<br>• Understanding of AMS in low- and middle-income context<br>• Understanding of pharmacy skills within global health<br>• Leadership skills<br>• Greater understanding of international development and health partnership principles<br>• Research skills, gathering data and evaluating outcomes<br>• Project management skills<br>• Understanding of how to apply behaviour change<br>• Opportunity to form new partnerships and link with others in the field<br>If you wish to add more information, you can use this comment box.	Included	
2. During CwPAMS did you have a dedicated/allocated lead/formal role in delivering any of the identified objectives or monitoring and learning indicators for CwPAMS?<br>Yes/No	Included	
3. Which indicator or activity do you have the lead for delivering?	Included	
4. Have you completed the following before and after June 2019:<br>• 360 assessment questionnaire<br>• NHS healthcare leadership model self-assessment<br>• Any other leadership self assessment<br>• A project management course<br>• Myers–Briggs Type Indicator Questionnaire<br>• Any other personality type indicator questionnaire<br>• Had a formal/semi formal discussion<br>• Written a project plan	Included	

• Formally led a project or project deliverable (ie. you were responsible for delivering a project and reporting the findings formally<br>• Led a quality improvement project<br>Use the following section to provide further information		
5. Do you currently use social media for professional activities?<br>Yes fully<br>Yes somewhat<br>Not at all	Exclude	Not the only form of communication. Does not directly reflect leadership. Personal preferences.
6. What would you say have been the key benefits to your NHS organisation through your participation in the fellowship	Included	
7. I felt the fellowship has been worthwhile/not worthwhile because....	Included	
8. Please state how much you agree with the following items:<br>**Confidence**<br>• I am confident in my ability to manage myself in a clinical environment.<br>• I am confident in my abilities to work independently when necessary.<br>• I am confident in my ability to deal with the unexpected.<br>• I am confident in my ability to be adaptable and innovative as a leader.<br>• I am confident in my ability to adapt and be flexible CLINICALLY.<br>• I am confident in my ability to adapt and be flexible IN GENERAL.<br>• I am confident in my ability to find solutions despite limited resources.<br>• I am confident in my ability to apply clinical skills to another context.<br>• I am confident in my work.<br>**Life Satisfaction**<br>• In most ways my life is close to my ideal.<br>• The conditions of my life are excellent<br>• I am satisfied with my life.<br>• So far I have gotten the important things I want in life.<br>• If I could live my life over, I would change almost nothing.<br>• Taking everything into consideration, I am satisfied with my job.<br>**Teaching**<br>• I am confident in my ability to teach others	Included confidence and teaching from MOVEIT tool questions<br><br>Excluded life satisfaction from<br>questions from analysis	Participants question why life satisfaction questions were asked as they did not seem relevant during a pandemic and could not be compared to baseline.

Feel free to add comments using this section:

9. In the last month…..<br>**Cultural**<br>• I demonstrated a good awareness about how culture influences health.<br>• I frequently demonstrated cultural sensitivity.<br>• I was constantly conscious of culture when working with patients.<br>**Adapting Communication**<br>• I changed the way I speak so that somebody can understand me (e.g. purposely spoke slower and clearer).<br>• I changed the way I communicate to make it more contextually appropriate (e.g., to make it more culturally appropriate).<br>• I frequently relied on my non-verbal communication (e.g. hand gestures).<br>**Teaching**<br>• I demonstrated I'm a good teacher.<br>• I adapted the way I teach to make it better for the learner.<br>**Difficult Communication**<br>• I demonstrated that I am skilled in challenging conversations, even in high pressure situations.<br>• I demonstrated that I am able to manage difficult people effectively.<br>• I frequently dealt with difficult people.<br>**Behaviour Change**<br>• I am able to empower patients to help themselves.<br>• I am able to empower colleagues to help themselves.<br>• In my work I have demonstrated skills in changing colleagues' behaviour.<br>• In my work I have demonstrated skills in encouraging and supporting patients to change behaviour.<br>**Management**<br>• I allocated tasks.<br>• I co-ordinated colleagues.<br>• I demonstrated I am able to plan and organise.<br>**Attitude to work**<br>• I was frequently proactive at work (e.g. used my initiative, got on with things, thought on my feet).	Included teaching, behaviour change, management, attitude to work and difficult communication from MOVEIT tool questions<br><br>Excluded cultural and adapting communication from MOVEIT tool questions.	Not all participants were in LMIC placements or exposed to experiences to be able to answer these questions. Participants did not find these questions relevant

• I demonstrated that I am able to cope in work (e.g. able to deal with stress).<br>• I demonstrated that I am particularly good at working as part of team.<br>• I demonstrated I'm good at dealing with the unexpected.<br>• I frequently had to find solutions despite limited resources.<br>• I demonstrated I am able to find solutions despite limited resources.<br>Please share further details:	
10. Reflecting over the last 12 months, state how much you agree with the following:<br><br>• I undertake more MDT work compared to a year ago<br>• I am more likely to work with other disciplines on a regular basis<br>• I have worked across disciplines in delivering AMS/IPC<br>• I find myself working more with different professional group<br>• I am more confident to approach people I have never worked with before compared to a year ago<br>• I am happy to work with / approach people who work outside the NHS in order to collaborate compared to a year ago<br>• I have started managing a new aspect of service<br>• I recognise the need to review / manage /introduce a new aspect of service<br>• I have become more involved in research<br>• I have made changes to the way in which I work as a team<br>• I have made changes to the way in which I engage with others in the work environment<br>• I have made changes to the way in which I teach<br>• I have made changes to the way in which I engage with the wider Trust/organisation<br>• I have made changes to the way I practice<br>• I have made changes to the way in which I engage with others outside a work environment<br>Please share further information on the changes seen:	Included

11. Over the past 12 months (June 2019 – June 2020), please indicate which of the following you have undertaken and if these were a new professional experience for you, i.e. you haven't previously done these things before? (check that apply)<br>• Publication / presentation of work at a conference<br>• Publication of work for a journal<br>• Publication / presentation of work within Trust setting<br>• Promotion of work on social media<br>• Collaboration on work within your Trust<br>• Collaboration on work at a regional / local level (outside your Trust)<br>• Collaboration on work at a National level<br>• Collaboration on work at an international level<br>• I have utilised a mentor<br>• I have become a mentor<br>• I have changed job role<br>• I have had a promotion<br>• I have undertaken Faculty assessment<br>• I have written a business case<br>• I have been involved in a healthcare partnership as described by Tropical Health Education Trust (THET) please see https://www.thet.org/our-work/health-partnership-scheme/<br>• I have undertaken an audit<br>• I have undertaken a project with a focus on quality improvement<br>• I have undertaken teaching<br>• I have enrolled or undertaken a leadership course<br><br>Other (please specify):	Included	
12. My experiences over the past 12 months have influenced the following improvements to existing services or new areas of work…. Please complete text boxes and provide any other details	Included	
13. What experiences do you think have influenced you to do / undertake these new challenges or what do you think was the key driver for this? Please complete text box and provide any other details	Excluded from analysis	Comments used to understand of result in discussion
14. Over the past 12 months, do you feel you have changed the way in which you shape / deliver the AMS strategy in your Trust and give examples	Excluded	Irrelevant for the purposes of this paper which consider leadership

of how? Please complete text box and provide any other details:		skills. Not all participants working in the arena of AMS in the UK
15. Have you increased your capacity within your team for AMS activities in the last 12 months? Yes/No	Excluded	
16. How well do you believe you coped in April and May with the COVID19 outbreak? Likert scale very well to not very well	Included	
17. What aided your coping mechanisms?	Excluded from analysis	Comments used to understand of result in discussion

Participant ID details. We need to be able to link your answers on this survey with answers on surveys in the future or past. Please share your email address so we can do that if you do not want to share your email address for this purpose, please generate a code by entering in the following in this order: The 3rd letter of your first name*the 4th letter of your first school*the first letter of your mother's first name*the date that you were born i.e., the 'day of the month from 01 to 31*the second letter of your last name. This should be a 6 figure string of letters and numbers that allows us to anonymously identify you

18. Gender	Included	
19. Age	Included	
20. Job title		
21. Employment status (before trip)	Excluded	Irrelevant for the purpose of this paper
22. In what sector is your primary role (i.e > 50%)	Included	
23. Years since registration	Included	
24. Nationality	Excluded	
25. Name of partnership	Excluded	
26. Before CwPAMS, have you previously participated in an international project or travelled to LMIC to volunteer/work? (please provide additional information) Yes/No	Included	
27. Did you use any annual leave for the trip(s)?	Excluded	Irrelevant for the purpose of this paper
28. How many in-country visits did you make during CwPAMS?	Excluded	
29. What was your primary motivation for participating in CwPAMS?	Excluded	
30. Did the following support your trip? Colleagues Deanary Line Manager Trust	Excluded	
31. Additional Post Project Questions • I would feel comfortable working outside of my competence	Exclude	Irrelevant for the purpose of this paper

• I would feel comfortable working in a high risk situation<br>• I think I will make a major difference to the host country within my trip<br>• I have an excellent understanding of my current professional knowledge e.g. I understand how much I currently know and what I need to work on in the future<br>• I felt engaged with the project throughout<br>• I learnt the host language<br>• I felt my skills were best utilised e.g. my skills were effectively utilised in the host country<br>• I interacted with more patients each day than I would in the UK<br>• I experienced a greater variety of conditions than I would in the UK<br>• I copied the behaviours of the staff in the host country e.g. agreed with and internalised lots of the knowledge, attitudes, skills and behaviours of other staff in the host facility<br>• At least once, I was aware of my opinions or perspectives changing in a significant way<br>• It was easy to accommodate the experiences I had into my own view of reality<br>• I understood the local context e.g. culture, customs, hierarchies, power dynamics		
32. The skills and knowledge I gained during my fellowship.... (Please check all applicable)<br>• are useful at the current stage in my career<br>• are applicable to my UK position<br>• None of the above	Included	
33. I felt the work on the project was...<br>• Too easy, repetitive or boring<br>• Challenging but achievable<br>• Overwhelming, beyond my capacity and frustrating<br>• None of the above	Excluded	Irrelevant for the purpose of this paper.<br>Does not directly reflect leadership.<br>Personal perspective.
34. What support did you have access to? Please check all applicable<br>• UK Mentor (directly linked to CwPAMS partnership)	Included	Acknowledge in the introduction

• UK mentor (not linked to global health)<br>Mentor assigned through CPhOGHFellowship<br>• Mentor in LMIC<br>• Supervision/support from other volunteers in LMIC (i.e. linking of junior and senior volunteers)<br>• Supervision/Support from local staff in LMIC<br>• Support from other CwPAMS volunteers<br>• Formal support structure in LMIC (e.g. access to HR)<br>• Support from volunteers working on another project (in country)<br>• Frequent feedback from a local senior colleague<br>• Frequent feedback from a western senior colleague<br>• Support from CPA or THET<br>• None of the above		
35. Generally I felt the experience of participating in a global health fellowship was...	Included	
36. About the LMIC visit<br>• Length of stay<br>• Country visited<br>• Town/City visited<br>• Type of facility/placement (e.g. rural hospital, regional referral hospital, community work)	Included the length of stay and country visited<br><br>Excluded town/ city visited and type of facility	Town and city visited and type of facility irrelevant for the purpose of this paper
37. Thinking about your CwPAMS project, please state how much you agree with the following statements:<br>• I felt engaged with the project throughout<br>• I learnt the host language<br>• I felt my skills were best utilised e.g. my skills were effectively utilised in the host country<br>• I interacted with more patients each day than I would in the UK<br>• I experienced a greater variety of conditions than I would in the UK<br>• I copied the behaviours of the staff in the host country e.g. agreed with and internalised lots of the knowledge, attitudes, skills and behaviours of other staff in the host facility<br>• At least once, I was aware of my opinions or perspectives changing in a significant way<br>• It was easy to accommodate the experiences I had into my own view of reality<br>• I understood the local context e.g. culture, customs, hierarchies, power dynamics<br>• To lead and have responsibility	Excluded	Irrelevant for the purposes of this paper. Question focusses on CwPAMS

• To visit more than one health facility<br>• To explore life outside of the hospital and immerse yourself in local culture<br>• None of the above		
38. What opportunities were available to you during your placement?<br>• To lead and have responsibility<br>• To visit more than one health facility<br>• To explore life outside of the hospital and immerse yourself in local culture<br>• None of the above	Excluded	
39. Which of the following are correct about the local staff you met on your placement? (Please check all applicable)<br>• They were under time pressures<br>• Many left or moved facilities within my stay<br>• I felt encouraged by them<br>• I had a local role model<br>• I experienced communication difficulties<br>• I engaged with them frequently<br>• They had adequate financial and human resources<br>• They were critical of volunteers and the project<br>• Hospital leaders were engaged with the project<br>• I engaged frequently with local staff<br>• I have stayed in touch with many of them<br>• There was frequently a more clinically knowledgeable person<br>• working alongside me<br>• There was frequently a more knowledgeable person (about local culture) working alongside me<br>• Many have adopted some of my the skills, knowledge and attitudes and used this in their practice<br>• It was obvious that we had many shared values<br>• None of the above	Excluded	Irrelevant for the purpose of this paper. Does not reflect leadership.
40. Did you experience any of the following as a result of your placement? (Please check all applicable)<br>• Health consequences (injuries, illness etc.)<br>• Loss of earnings (for time away)	Excluded	Irrelevant for the purpose of this paper

• Loss of pension or other employee benefits<br>• Exposure to corruption<br>• None of the above		
41. How many other projects were working in the healthcare facility?	Excluded	Participants were not always aware of the answer to this question having limited time in country.
42. I critically reflected upon my experience (Please check all applicable)<br><br>• During my placement<br>• Upon return from my placement<br>• Formally<br>• Informally<br>• None of the above	Included	
43. Generally I felt the experience was...	Included	
44. Do you have any comments regarding the questions in this section?	Included	Qualitative data
45. Thank you very much for completing the questionnaire. Do feel free to use this section to provide additional comments	Included	Qualitative data

Table S3. Assessment of CPhOGH Fellows Leadership skills by Senior Leaders.

Question	Included/ Excluded	Rational
1. In order to proceed with this questionnaire, we ask that you confirm the following:<br>• I have worked with the Fellow for whom this feedback is for, at least a year<br>• I hold a more senior role to the above Fellow	Included	
2. The questionnaire is an important part of the Commonwealth Pharmacy Association's (CPA) service provision for the Commonwealth Partnerships for Antimicrobial Stewardship (CwPAMs) and CPhOGHFellowship.<br>By proceeding to the next page:<br>• I consent to CPA collecting and using the information about me that I provide for the purposes of the survey<br>• I have read, understand and agree to the information provided above		
3. What is your core profession?	Included	
4. What is your relationship to the Fellow?	Included	
5. What code has the Fellow provided you with?	Excluded	
**For question 6–11 senior colleagues were asked to assess the fellow's performance pre and post CPhOGH Fellowship using a Performance rating (0 = unable to comment; 1= essential; 2 = proficient / established; 3 = strong / excellent; 4 = exceptional /exemplary)**		
6. **Team Work**<br>• The fellow listens attentively to other team members and values their suggestions<br>• The fellow is an established and effective member of a multidisciplinary team<br>• The fellow is consulted for advice which requires their in-depth professional expertise<br>• The fellow consistently works effectively across boundaries to build relationships and share information, plans and resources.<br>• Please provide further context or example of the fellow demonstrating practice in this area:	Included	
**7. Vision, Motivate and Develop Capability**<br>• The fellow creates a clear vision of the future for his/ her team in accordance with the organisations vision.<br>• The fellow often looks for opportunities to develop him/herself and learn things outside his/her comfort zone.<br>• The fellow often provides constructive feedback to his/her team to help them focus on the right area in order to develop professionally<br>• The fellow is involved in strategy planning<br>• Please provide further context or example of the pharmacist demonstrating practice in this area:	Included	

**8. Influencing for results**<br>• The fellow is able to share issues and information to help others understand his/her point of view.<br>• The fellow tailors his/her communication group or presentation according to the audience or stakeholder.<br>• The fellow directly or indirectly links different working groups across the organisation to achieve a common goal.<br>• The fellow gains reputational influence by sharing experience and best practice nationally<br>• Please provide further context or example of the fellow demonstrating practice in this area:	Included
**9. Inspiring shared purpose**<br>• The fellow demonstrates the characteristics of a role model of the Pharmacy profession.<br>• The fellow inspires others, even when they are under pressure, by helping them to focus on the value of their contribution<br>• The fellow supports his/her colleagues to keep challenging others cohesively to achieve a shared purpose.<br>• Please provide further context or example of the fellow demonstrating practice in this area:	Included
**10. Managing Change**<br>• The fellow is able to adapt to different way of working especially in times of crisis<br>• The fellow consistently reflects on his/ her service and manages processes of change or service improvement<br>• The fellow applies a behaviour change approach in change management or education and training<br>• Please provide further context or example of the fellow demonstrating practice in this area:	Included
**11. Innovative working and practice**<br>• The fellow strives to improve his/her service within the limitation of resources.<br>• The fellow is able to analyse essential data and utilise the results to improve services.<br>• The fellow is able to identify the key stakeholders and understand their agenda.<br>• The fellow effectively strives to improve quality within limitations of service<br>• The fellow recognises and implements innovation from the external environment.<br>• The fellow takes the lead to ensure innovation produces demonstrable improvement<br>• Please provide further context or example of the pharmacist demonstrating practice in this area:	Included
12. Do you think the fellow will be ready for a more senior role / take up additional challenges (within their current role) in the next 6-12 months?	Included

13. What impact do you think the fellowship and involvement in the global health project (CwPAMS) has had on the fellow professionally?	Included
14. Have you observed other changes / differences in the fellow throughout his/her CPhOGH fellowship?	Included
15. Would you recommend the CPhOGH fellowship to other pharmacists in your department?	Included
16. How well do you believe the fellow coped in April and May with the COVID19 outbreak?	Included

Table S4. Additional activities of the CPhOGH Fellows during the Fellowship year.

Activity	Additional details
Journal Publications	JAC AMR<br>Pharmaceutical Journal
Conference representation<br>Presentations/posters	RPS; BSAC; UKCPA PIN; Disaster international; THET; IPS; SAPG; Public health festival; FIP
Commonwealth Pharmacists Association support	Covid19 webinar series<br>Video on hand gel production
Research	Behaviour change and AMS
Academic teaching	
Presentation to Trust Boards	
Podcasts/Radio	Pharmacy in Practice<br>Pharmacist diaries<br>Local radio
Blogs	Fleming fund<br>RPS<br>Trust intranet<br>Global Health
Community engagement	Twinning of primary schools<br>Crowd funding
National AMS messaging Campaign	World Antibiotic Awareness Week

Table S5. Themes from the additional activities of the CPhOGH Fellows.

Development of leaders	Behaviour Change	Working in resource limited settings	Development of networks	Understanding of working in LMICs
Negotiation skills	Approach to education and training	Work beyond traditional boundaries	Academia	How to build cohesive relationships
Communication skills	To work differently and innovatively	Frugal innovation "improvements don't always require money"	Research	Greater understanding of public health issues
Difficult conversations	Reflection	Expect the unexpected (COVID-19)	Increased exposure for local Trusts	
Caring for colleagues		How collaborative efforts can provide further benefits to all	National	
Project management skills		Drug shortages and other supply issues	International	

Table S6. Demographic of the CPhOGH Fellows.

	Frequency (*n* = 16, unless otherwise stated*)	Percentage (%)
**Gender**		
Male	3	19%
Female	13	81%
**Age (n = 15)***		
20-30	2	13%
31-40	7	47%
41-50	5	33%
51-60	1	7%
**Years Since Registration (n = 15)**		
< 5 years	2	13%
5-10 years	2	13%
11-20 years	7	47%
21-30 years	3	20%
31-40 years	1	7%
**Sector or Primary Role**		
Acute Trust	12	75%
Community	1	6%
Academia	3	19%
**Global Health Experience**		
Yes	5	31%
No	11	69%

Table S7. Statements for the components of MOVE-iT.

Statements from MOVE-iT	Pre CPhOGH (n= 16)							Post CPhOGH (n=16)						
	Strongly Agree	Agree	Somewhat agree	Neutral	Somewhat disagree	Disagree	NA	Strongly Agree	Agree	Somewhat agree	Neutral	Somewhat disagree	Disagree	NA
**Confidence**														
I am confident in my ability to manage myself in a clinical environment.	6	6	3	1				11	4	1				
I am confident in my abilities to work independently when necessary.	8	8						13	3					
I am confident in my ability to deal with the unexpected	5	4	7					10	6					
I am confident in my ability to be adaptable and innovative as a leader	5	3	7	1				9	5	2				
I am confident in my ability to adapt and be flexible CLINICALLY.	4	6	5	1				6	8	2				
I am confident in my ability to adapt and be flexible IN GENERAL.	6	7	3					9	6	1				
I am confident in my ability to find solutions despite limited resources.	3	5	7	1				9	5	2				
I am confident in my ability to apply clinical skills to another context.	3	4	7	1	1			9	5	2				
I am confident in my work.	5	7	4					10	5	1				
**Teaching**														
I am confident in my ability to teach others	3	8	5					8	7	1				
I demonstrated I'm a good teacher.	4	7	2	3				6	8		2			
I adapted the way I teach to make it better for the learner.	5	6	1	3	1			10	5	1				
**Difficult Communication**														

I demonstrated that I am skilled in challenging conversations, even in high pressure situations.	2	7	4	2	1			9	5	1	1			
I demonstrated that I am able to manage difficult people effectively.	1	4	4	3	3	1		8	5	2	1			
I frequently dealt with difficult people.	3	4	2	1	3	3		6	4	5			1	
**Behaviour Change**														
I am able to empower patients to help themselves.	2	3	7	3			1	5	7	2				2
I am able to empower colleagues to help themselves.	4	5	5	2				9	7					
In my work I have demonstrated skills in changing colleagues' behaviour.	3	5	1	4	3			8	5	3				
In my work I have demonstrated skills in encouraging and supporting patients to change behaviour.	2	5	5	2			2	3	9	2				2
**Management**														
I allocated tasks.	5	6	3		2			11	4	1				
I coordinated colleagues.	5	5	4	1			1	11	4	1				
I demonstrated I am able to plan and organise.	6	7	3					10	5	1				
**Attitude to work**														
I was frequently proactive at work (e.g. used my initiative, got on with things, thought on my feet).	8	7		1				11	4	1				

I demonstrated that I am able to cope in work (e.g. able to deal with stress).	9	5	2					9	7					
I demonstrated that I am particularly good at working as part of team.	10	4	2					9	7					
I demonstrated I'm good at dealing with the unexpected	7	6	3					9	6	1				
I frequently had to find solutions despite limited resources.	2	7	5	1	1			7	5	3				1
I demonstrated I am able to find solutions despite limited resources.	2	6	5	2	1			8	5	2	1			

Figure S1: Average Leadership Dimension Score per CPhOGH Fellows

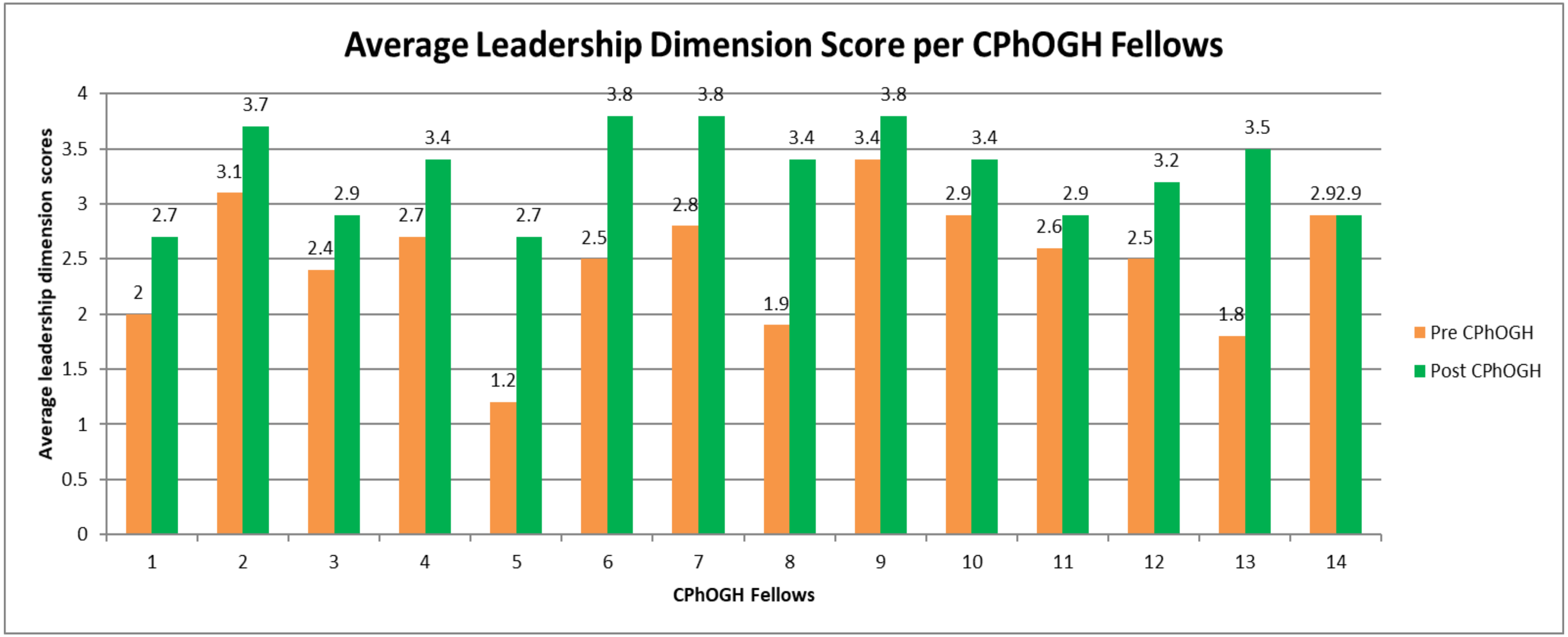

Average leadership dimension scores per fellow from senior colleague questionnaire
0 = unable to comment; 1 = essential; 2 = proficient / established; 3 = strong / excellent; 4 = exceptional / exemplary
